# Cerebral vasoreactivity is not impaired in patients with severe sepsis

**DOI:** 10.1186/cc9739

**Published:** 2011-03-11

**Authors:** S Szatmári, Z Fülep, P Sárkány, C Antek, P Siró, C Molnár, B Fülesdi

**Affiliations:** 1 University of Debrecen, Hungary

## Introduction

In a previous report it was observed that acetazolamide-induced cerebrovascular reactivity is impaired in patients with sepsis-associated encephalopathy without organ dysfunction [1]. The aim of the present work was to assess whether patients suffering from severe sepsis also have these impaired cerebrovascular responses.

## Methods

Patients fulfilling the criteria of clinical sepsis and showing at least two organ dysfunctions other than the brain were included (*n *= 14). Nonseptic persons without previous diseases affecting cerebral vasoreactivity served as controls (*n *= 20). Transcranial Doppler blood flow velocities were measured at rest and at 5, 10, 15 and 20 minutes after intravenous administration of 15 mg/kg BW acetazolamide. The time course of the acetazolamide effect on cerebral blood flow velocity (cerebrovascular reactivity) and the maximal vasodilatory effect of acetazolemide (cerebrovascular reserve capacity (CRC)) were compared among the groups.

## Results

Mean blood flow velocity in the middle cerebral artery was lower (41.7 ± 13.3 cm/second) in septic patients at rest than in controls (58.2 ± 12.0 cm/second, *P *< 0.01). Pulsatility indices were higher among septic patients at rest (1.56 ± 0.79) than in controls (0.85 ± 0.20, *P *< 0.01). Assessment of the time course of the vasomotor reaction showed that patients with sepsis reacted in similar fashion and extent to the vasodilatory stimulus than did control persons. When assessing the maximal vasodilatory ability of the cerebral arterioles to acetazolamide during vasomotor testing, we found that patients with sepsis reacted to a similar extent to the drug than did control subjects (CRC controls:46.2 ± 15.9%, CRC SAE: 63.2 ± 28.4%).

## Conclusions

Cerebral vasoreactivity to acetazolemide is not impaired in patients with severe sepsis. Our data suggest that the reaction of the cerebral arterioles to vasoactive stimuli changes along with the severity of the septic process.
